# Autophagy promotes the survival of dormant breast cancer cells and metastatic tumour recurrence

**DOI:** 10.1038/s41467-018-04070-6

**Published:** 2018-05-22

**Authors:** Laura Vera-Ramirez, Suman K. Vodnala, Ryan Nini, Kent W. Hunter, Jeffrey E. Green

**Affiliations:** 1 Laboratory of Cancer Biology and Genetics, National Cancer Institute, National Institutes of Health, Bethesda, MD 20892 USA; 2 Present Address: Department of Genitourinary Medical Oncology, The University of Texas MD Anderson Cancer Center, Houston, TX 77030 USA

## Abstract

Cancer recurrence after initial diagnosis and treatment is a major cause of breast cancer (BC) mortality, which results from the metastatic outbreak of dormant tumour cells. Alterations in the tumour microenvironment can trigger signalling pathways in dormant cells leading to their proliferation. However, processes involved in the initial and the long-term survival of disseminated dormant BC cells remain largely unknown. Here we show that autophagy is a critical mechanism for the survival of disseminated dormant BC cells. Pharmacologic or genetic inhibition of autophagy in dormant BC cells results in significantly decreased cell survival and metastatic burden in mouse and human 3D in vitro and in vivo preclinical models of dormancy. In vivo experiments identify autophagy gene autophagy-related 7 (ATG7) to be essential for autophagy activation. Mechanistically, inhibition of the autophagic flux in dormant BC cells leads to the accumulation of damaged mitochondria and reactive oxygen species (ROS), resulting in cell apoptosis.

## Abstract (web summary)

Highly metastatic dormant cancer cells contribute to breast cancer recurrence, but the underlying mechanism is less understood. Here, the authors show that dormant breast cancer cells depend on autophagy to ensure their long term survival and distant recurrence

## Introduction

Ninety percent of BC-related deaths are due to metastatic disease^1^. Despite metastasis being the leading cause of BC-related mortality, the molecular mechanisms of metastatic progression remain poorly understood^2^. Although most patients do not present with overt metastases at diagnosis, a significant number succumb to disseminated disease years after the removal and treatment of the primary tumour. Disseminated tumour cells (DTCs) have frequently been observed at early stages of BC suggesting that late recurrence of BC may result from DTCs that have remained quiescent for decades^3,4^. Signals that trigger the outgrowth of dormant cancer cells remain largely unknown, although the tumour microenvironment plays a critical role in this process^5–7^.

We previously developed and validated in vitro and in vivo model systems to study BC dormancy^8–10^. Briefly, the D2A1 and D2.0 R tumour cell lines (derived from murine mammary hyperplastic alveolar nodules^11,12^) form primary tumours when injected into the mammary fat pad of mice and disseminate to the lungs. D2A1 cells form macrometastases in the lungs within ~1–3 weeks. In contrast D2.0 R cells remain dormant at the metastatic site for about 4 months before forming relatively few lung metastases^13^. The 3D in vitro system has been shown to be predictive of the dormant or proliferative phenotype of several mouse and human BC cell lines^8^. D2.0 R and MCF-7 cells remain quiescent on basal membrane extract (BME) matrices for 12 days whereas the highly metastatic D2A1, MDA-MB-231 and 4T1 cells spontaneously outbreak into a proliferative state between day 1 and 6 of culture on BME^8^. These studies demonstrated that changes in the microenvironment, including exposure to collagen 1 (COL1) or fibronectin, induce the dormant-to-proliferative switch of D2.0 R cells^8,10^. In vivo studies are consistent with these in vitro findings, where lung fibrosis induced by the intranasal instillation of a transforming growth factor beta (TGFβ) expressing adenoviral vector drives the proliferative outbreak of otherwise dormant D2.0 R cells when seeded to the lungs by tail vein injection^9^. We have previously shown that the dormant-to-proliferative switch of D2.0 R cells requires the activation of integrin β1 receptor and downstream signalling through focal adhesion kinase (FAK), Src, ERK1/2 and myosin light chain kinase (MLCK) leading to actin stress fibre formation^8,9^. Moreover, the pharmacological inhibition of Src and MEK prevented the proliferative outbreak of dormant D2.0 R cells^14^ in vivo.

Little is understood about the processes associated with the survival of disseminated dormant tumour cells. Although autophagy has been proposed as a potential mechanism promoting dormant cancer cell survival, few studies have addressed this experimentally^15–18^. Autophagy is an evolutionarily conserved mechanism of cell survival activated in response to metabolic stress to degrade organelles, misfolded proteins and portions of the cytosol to ensure proper energy balance under nutrient deprivation conditions and to recycle dysfunctional organelles and macromolecules^19^.

In this study, we demonstrate that pharmacologic or genetic inhibition of autophagy greatly impairs the survival of dormant BC cells in vitro and in vivo, but has minimal effect on metastatic growth once dormant cells have transitioned to a proliferative state. Moreover, inhibition of autophagy results in the accumulation of damaged mitochondria and oxidative stress that drives apoptotic cell death. Inhibition of autophagy may therefore be a potential mechanism to eliminate dormant tumour cells and prevent recurrence of BC.

## Results

### Solitary dormant tumour cells are autophagic

**Fig. 1:**
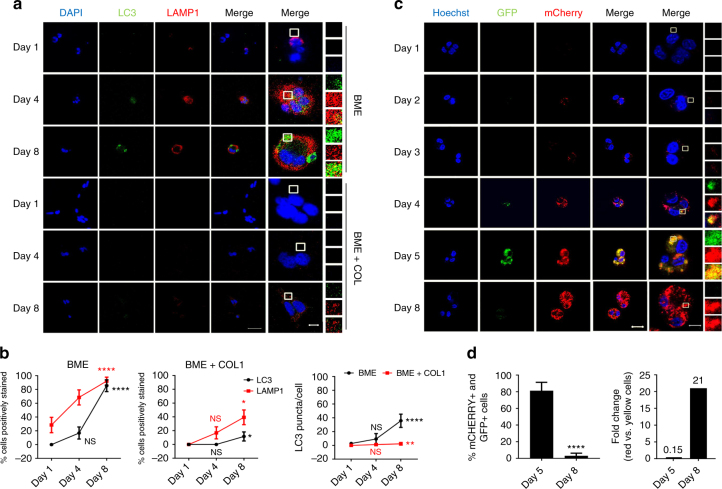
D2.0 R cells activate autophagy upon entering a dormant state. Representative images are shown for **a** Immunofluorescent staining of lysosomal (LAMP1, red) and autophagic (LC3, green) markers of D2.0 R cells in BME (upper panels) and BME + COL1 (lower panels) with **b** Quantification of the percentage of cells exhibiting LC3 positive puncta and LAMP1 staining out of the total number of cells analysed (mean ± s.e.m, n = 30–42 cells. Comparisons are relative to Day 1 by Kruskal–Wallis, Dunn’s post test. *P ≤ 0.05; ****P ≤ 0.0001) and the average number of LC3 puncta per cell (Comparisons by Kruskal–Wallis, Dunn’s post test. **P ≤ 0.01 and ****P ≤ 0.0001). **c** D2.0 R cells transfected with the mCherry-GFP-LC3 reporter show activation and completion of the autophagic cycle (red fluorescence only) when plated in BME matrices for 8 days. **d** The graphs represent the percentage of cells with double-positive puncta (mCherry^+^ and GFP^+^) (autophagosomes) out of the total number of cells analysed (left graph) (mean ± s.e.m, *n* = 35–39 cells). Comparisons by Kruskal–Wallis, Dunn’s post test. ****P ≤ 0.0001) and the rate of conversion from cells exhibiting yellow puncta to red-only puncta expressed as fold change (right graph). Scale bars are 50 and 10 μm for **a** and 20 and 10 μm for **b**

To investigate the occurrence of autophagy in dormant breast tumour cells, we analysed the expression pattern of Microtubule-associated protein 1 A/1B-light chain 3 (MAP1LC3, also known as LC3) and Lysosomal-associated membrane protein 1 (LAMP1) over time in D2.0 R cells on BME (cells remain dormant) and BME plus COL1 matrices (which induces proliferation of the dormant cells)^8^ (Supplementary Fig. 1). Consistent with activation of autophagy, D2.0 R cells in BME showed increased expression of LAMP1 and LC3 over time. In contrast, D2.0 R cells in BME plus collagen 1 (COL1) exhibited much lower levels of LAMP1 and LC3 staining throughout the 8-day time course (Fig. 1a, b). Validation of autophagy in D2.0 R cells on BME was demonstrated using a tandem fluorescent-tagged LC3B reporter containing mCherry and EGFP (mCherry-EGFP-LC3) that allows real time monitoring of autophagic flux in live cells^20^ (Supplementary Fig. 2). Yellow LC3 puncta (early autophagosomes not yet fused with lysosomes) were detected in 81.25% of dormant D2.0 R cells at Day 5 of culture. This percentage dropped down to 3.12% at Day 8 (Fig. 1c, d), at which time 68.75% of dormant D2.0 R cells exhibited red-only fluorescent puncta (mature autolysosomes) (Fig. 1c). We conclude that the non-proliferating BC cells are autophagic while in a dormant state.

### Autophagy inhibition decreases viability of dormant cells

**Fig. 2:**
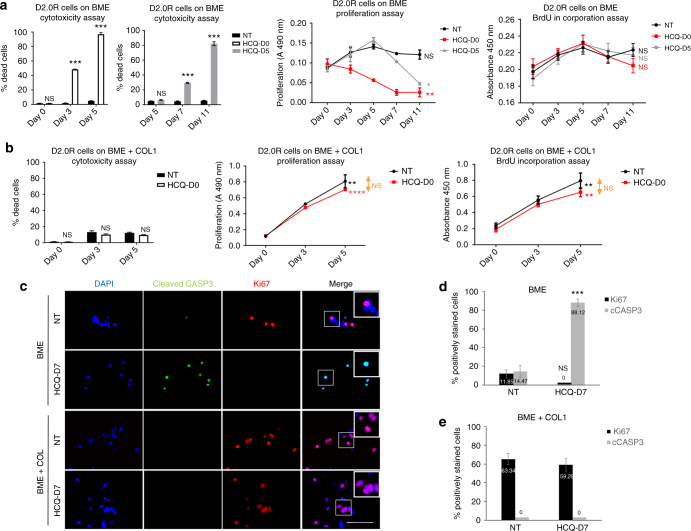
Inhibiting autophagy reduces the viability of D2.0 R dormant cells. Figure shows data of one out of three independent experiments done in triplicate with equivalent results for each assay. **a** 50 µM Hydroxycholoroquine (HCQ) induced a significant reduction in the proportion of viable D2.0 R cells on BME, as determined by cytotoxicity assay (mean ± s.e.m, *n* = 3 wells. Comparisons by unpaired two-sided *T*-test. ***P ≤ 0.0001), proliferation assay (mean ± s.e.m, *n* = 3 wells. Comparisons at day 11 by unpaired two-sided *T*-test. *P ≤ 0.05 and **P ≤ 0.01 relative to Day 0 for each group) and BrdU incorporation assay (mean ± s.e.m, *n* = 3 wells. Comparisons at Day 5 by unpaired two-sided *T*-test relative to Day 0 for each group) regardless of whether the treatment was or was not delayed after seeding the cells. **b** HCQ did not affect the numbers of viable D2.0 R cells on BME + COL, as determined by a cytotoxicity assay (mean ± s.e.m, *n* = 3 wells. Comparisons by unpaired two-sided *T*-test), proliferation assay (mean ± s.e.m, *n* = 3 wells. Comparison Day 11 vs. Day 0 by unpaired two-sided *T*-test. **P ≤ 0.01 and ****P ≤ 0.0001) and BrdU incorporation assay (mean ± s.e.m, *n* = 3 wells. Comparisons at Day 5 by unpaired two-sided *T*-test. **P ≤ 0.01 relative to Day 0 for each group). **c** Representative Ki67 (red) and cleaved caspase 3 (green) immunofluorescence of D2.0 R cells on BME (upper panels) and D2.0 R cells on BME + COL (lower panels), either treated with 50 µM HCQ or vehicle. Scale bar is 100μm. Proliferative and apoptotic indexes of D2.0 R on BME or BME plus COL1 were calculated as the percentage of cells expressing Ki67 or cleaved Casp 3, as shown in **d** for cells on BME (mean ± s.e.m, *n* = 159–173 cells. Comparison by Mann–Whitney *U*-test, two-sided. ***P ≤ 0.0001) and **e** for cells on BME + COL1 (mean ± s.e.m, *n* = 167–257 cells. Comparison by Mann–Whitney *U*-test, two-sided), respectively. HCQ-D0 cells treated with HCQ immediately after plating on Day 0, HCQ-D5 cells treated with HCQ after 5 days in culture, HCQ-D7 cells treated with HCQ after 7 days in culture, NT non-treated

To test the functional importance of autophagy in BC dormancy, D2.0 R cells were seeded on BME and treated with 50 μM Hydroxychloroquine (HCQ), an inhibitor of autophagy^21^, (based upon a dose-response analysis; see Supplementary Fig. 3a) either immediately or after 5 days in culture (Supplementary Fig. 4a). We observed a significant increase in the percentage of dead D2.0 R cells, regardless of when the treatment with HCQ was initiated. Likewise, the number of viable cells decreased across the 11 days of culture under the immediate or delayed treatment conditions (Fig. 2a). Similar results were observed when D2.0 R seeded on BME matrices were treated with other autophagy inhibitors, such as 3-Methyladenine (3-MA) or Bafilomycin (Supplementary Fig. 3b). Conversely, HCQ treatment did not affect the viability of proliferating D2.0 R cells on BME + COL1 (Fig. 2b). To visualise apoptotic and proliferative events under these conditions, cells were treated with HCQ at Day 7 of culture and stained for proliferation (Ki67) and apoptosis (cleaved Caspase 3 (cCASP3)) markers at Day 11 (Supplementary Fig. 4b). After treatment, the percentage of cCASP3-positive D2.0 R cells on BME significantly increased with respect to the non-treated control (Fig. 2c, upper panels and d). In contrast, the proliferative and apoptotic indexes of growing D2.0 R cells in BME plus COL1 were not affected by the treatment (Fig. 2c, lower panels, and e).

**Fig. 3:**
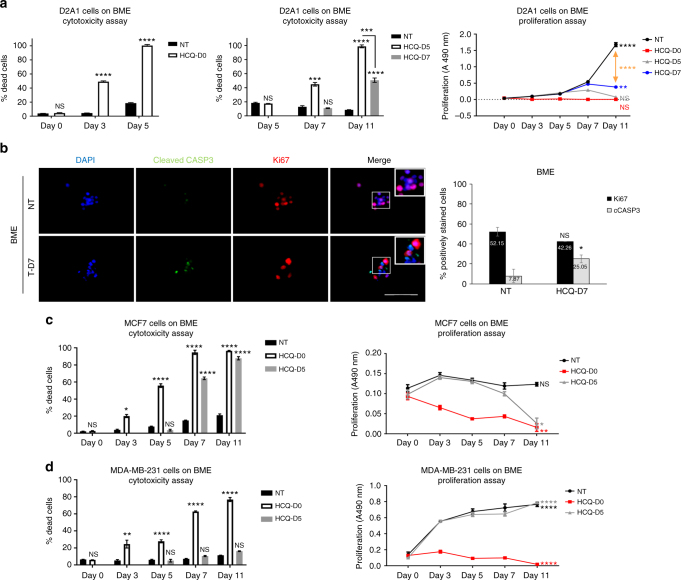
Inhibiting autophagy reduces proliferative switch in 3D culture. Figure shows data of one out of three independent experiments done in triplicate with equivalent results. **a** 50 µM Hydroxycholoroquine (HCQ) significantly reduced the proportion of viable D2A1 cells on BME during quiescence (treatment at day 0 (D0), left panel), which is attenuated when treated after the dormant-to-proliferative switch (day 5 (D5) or day 7 (D7), middle panel) as determined by cytotoxicity assay (mean ± s.e.m, *n* = 3 wells. Comparisons at day 5 (left panel) or day 11 (middle panel) by unpaired two-sided *T*-test. ***P ≤ 0.001 ****P ≤ 0.0001) and proliferation assays (right panel. Mean ± s.e.m, *n* = 3 wells. Comparisons at Day 11 by unpaired two-sided *T*-test. **P ≤ 0.01 and ****P ≤ 0.0001 relative to day 0 for each group). **b** Representative Ki67 (red) and cleaved caspase 3 (green) immunofluorescence of D2A1 cells on BME treated with 50 µM HCQ or vehicle (left panel), with quantification of markers at day 11 (right panel, mean ± s.e.m, *n* = 170–194 cells. (Mann–Whitney *U*-test, two-sided. *P ≤ 0.05). Scale bar is 100 μm. **c** HCQ significantly reduced viable MCF7 cells on BME (left panel, mean ± s.e.m, *n* = 3 wells. Comparisons by two-sided unpaired *T*-test (Day 0 and Day 3) or one-way ANOVA plus Bonferroni post test for day 5, day 7 and day 11 time points. **P ≤ 0.01; ****P ≤ 0.0001) and proliferation assays (right panel, mean ± s.e.m, *n* = 3 wells. Comparisons at day 11 by unpaired two-sided *T*-test. *P ≤ 0.05; **P ≤ 0.01 relative to day 0 for each group). **d** HCQ did not significantly reduce the number of MDA-MB-231 viable cells on BME when treated after the dormant-to-proliferative switch (left panel, mean ± s.e.m, *n* = 3 wells. Comparisons by unpaired two-sided *T*-test (day 0 and day 3) or one-way ANOVA plus Bonferroni post test for day 5, day 7 and day 11 time points. **P ≤ 0.01; ****P ≤ 0.0001) and proliferation assay (right panel, mean ± s.e.m, *n* = 3 wells. Comparisons at day 11 by unpaired *T*-test, two-sided. ***P ≤ 0.001; ****P ≤ 0.0001 relative to Day 0 for each group). HCQ-D0 cells treated with HCQ immediately after plating, HCQ-D5 cells treated with HCQ after 5 days in culture, HCQ-D7 cells treated with HCQ after 7 days in culture, NT non-treated

We tested the effect of autophagy inhibition on D2A1 cells seeded on BME matrices before and after their dormant-to-proliferative switch which occurs after 4–6 days of culture^8^ (Supplementary Fig. 1). We observed a significant increase in the percentage of dead cells when HCQ was added immediately after the cells were seeded on BME matrices (Fig. 3a). However, beginning treatment of proliferating D2A1 cells on day 7 resulted in a two-fold decrease in the percentage of dead cells compared to cells treated at day 5 of culture, before they undergo the dormant-to-proliferative switch (Fig. 3a).

Interestingly, the proliferation rate of D2A1 cells on BME treated from Day 7 of culture did not increase over time as much as their non-treated counterparts (Fig. 3a). This result raised the question of whether HCQ treatment reverted proliferating D2A1 cells into a quiescent state or, alternatively, whether the fraction of dead D2A1 cells resulting from HCQ treatment significantly reduced the growth kinetics of the entire cell population. To answer this question, we determined the proliferative and apoptotic indexes of D2A1 cells on BME treated with HCQ at Day 7 of culture. After 4 days of HCQ treatment the percentage of D2A1 cells expressing higher levels of cCASP3 significantly increased when compared with the untreated control. However, the proliferative index determined by ki67 staining was not different (Fig. 3b), confirming that surviving D2A1 cells continue proliferating at the same rate as their untreated counterparts.

Human BC MCF7 and MDA-MB-231 cells have been previously shown to exhibit comparable proliferative behaviour to D2.0 R and D2A (dormant vs. proliferative, respectively) in 3D culture (Supplementary Fig. 1) and in vivo^8^. To further investigate the role of autophagy in BC dormancy using human models, we seeded MCF7 and MDA-MB-231 cells on BME matrices and either treated the cultures immediately with HCQ or delayed treatment until day 5 (Supplementary Fig. 4a). HCQ significantly reduced the number of viable quiescent MCF7 cells whereas HCQ did not reduce the number of viable proliferative MDA-MB-231 cells, similar to the responses to HCQ observed for D2.0 R and D2A1 cells on BME, respectively (Fig. 3c, d). Collectively, these results demonstrate that mouse and human metastatic BC cells in a dormant state are sensitive to autophagy inhibition whereas proliferative cells are resistant.

### Lung metastatic burden is reduced by autophagy inhibition

Based on our in vitro results, we investigated the effect of inhibiting autophagy on metastatic outbreak in vivo, using our previously described D2.0 R TGFβ fibrotic lung dormant-to-proliferative switch model^9^ (Supplementary Fig. 5a). Otherwise dormant D2.0 R cells become proliferative when introduced to a fibrotic lung environment.

The administration of 50 mg/kg HCQ to animals nasally instilled with empty adenovirus (Ad-empty; inducing a non-fibrotic, dormancy-permissive lung microenvironment) consistently resulted in a significant decrease in the average metastatic burden compared to untreated mice (Supplementary Fig. 5b). In animals induced to develop lung fibrosis with adenovirus TGFβ^223/225^ that results in metastatic outgrowth of dormant cells, we observed a 36-fold decrease of total metastatic burden in HCQ-treated mice as compared to controls (Supplementary Fig. 5b; representative images in Supplementary Fig. 5c). Consistent with the in vitro data described above, treatment with HCQ shortly after injection of D2.0 R cells significantly reduced the metastatic burden in mice either with or without fibrosis, confirming the in vivo efficacy of inhibiting autophagy to reduce survival and metastatic outbreak of disseminated dormant BC cells.

### Autophagy blockade selectively targets dormant BC cells

**Fig. 4:**
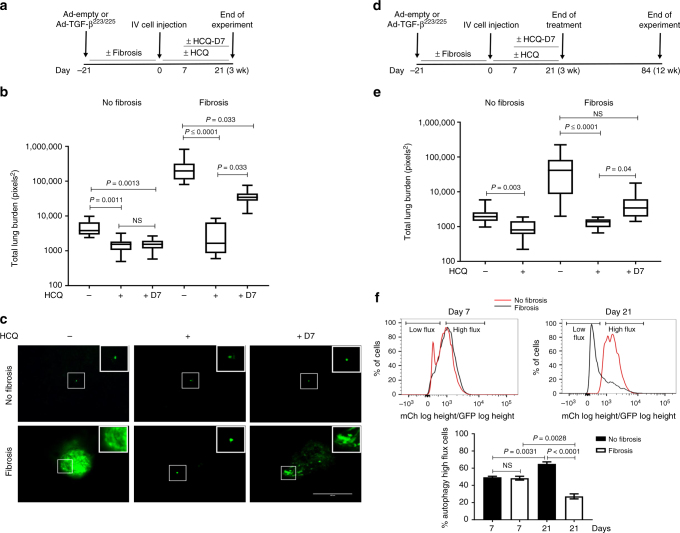
Delayed autophagy inhibition increases viable cells in fibrotic lungs. **a** Experimental design. **b** Total lung surface burden of CD1^nu/nu^ mice receiving Ad-empty (No fibrosis) or Ad-TGF-β^223/225^ (Fibrosis) and tail vein injections of 1 × 10^6^ D2.0 R GFP cells, followed by vehicle (−), 50 mg/kg body weight of HCQ 5 days a week for 3 weeks (+) or vehicle daily for 7 days followed by 50 mg/kg body weight of HCQ 5 days a week for additional 2 weeks (+D7) (mean ± s.e.m, *n* = 9–10 mice per group. Comparisons by Kruskal–Wallis, Dunn’s post test). **c** Representative images of single dormant cells and multicellular metastatic lesions expressing GFP in the lung from the experiment in **b**, scale bar is 400 μm. **d** Similar experiment to that in **a**, except that the time animals were followed after the cessation of treatment with HCQ to the final study endpoint that was extended for additional 9 weeks. **e** Total lung surface D2.0 R GFP cell burden from experiment **d** of CD1^nu/nu^ mice receiving Ad-empty (No fibrosis) or Ad-TGF-β^223/225^ (Fibrosis) and tail vein injections of 1 × 10^6^ D2.0 R GFP cells, followed by vehicle (−), 50 mg/kg body weight of HCQ 5 days a week for 3 weeks (+) or vehicle daily for 7 days followed by 50 mg/kg body weight of HCQ 5 days a week for additional 2 weeks (fibrosis group only) (+D7) (mean ± s.e.m, *n* = 9–10 mice per group. Comparisons by Kruskal–Wallis, Dunn’s post test). **f** High autophagy flux D2.0 R cells present in the lung of CD1^nu/nu^ mice receiving Ad-empty (No fibrosis) or Ad-TGF-^β223/225^ (Fibrosis) and tail vein injections of 1 × 10^6^ D2.0 R mCherry-GFP-LC3 cells after 7 or 21 days (mean ± s.e.m, *n* = 15 mice per group. Comparisons by Kruskal–Wallis, Dunn’s post test)

We next investigated whether administration of HCQ following the proliferative outbreak of dormant cells in a fibrotic environment would impact the metastatic burden in vivo (Fig. 4a). As expected, the administration of HCQ to animals instilled with Ad-empty resulted in a significant decrease of the average metastatic burden, regardless of when the treatment was initiated (Fig. 4b). However, in the fibrosis setting, autophagy inhibition after the proliferative lesions had developed (treatment-delay group) was remarkably less effective in reducing metastatic lesions, leading to a higher metastatic burden in the treatment-delay group as compared with the treatment group (Fig. 4b) (representative images of the metastatic lesions in Fig. 4c and Supplementary Fig. 6).

To assess the long-term effects of autophagy inhibition on disseminated BC cells, the experiment was extended for an additional 9 weeks following treatment cessation (Fig. 4d). The results recapitulated those obtained in the previous experiment, except that within the fibrosis setting the metastatic burden of control and treatment-delay groups was no longer significantly different (Fig. 4e). These data suggested that the surviving population of metastatic cells in a fibrotic environment after treatment with HCQ continued proliferating during the extended experimental time.

To further demonstrate the specificity of autophagy blockade in eliminating dormant BC cells, D2.0 R cells labelled with mCherry-EGFP-LC3 were tail vein injected in mice 3 weeks after nasal instillation with Ad-empty or Ad-TGFβ^223/225^ (Methods and Supplementary Fig. 7). Remarkably, most D2.0 R cells maintained high autophagic flux during the first seven days both in non-fibrotic and fibrotic lungs. However, after 21 days the percentage of high autophagy flux cells in the lungs of mice instilled with Ad-empty significantly increased compared to mice bearing fibrotic lungs (Fig. 4f). These data further confirmed that autophagy is a critical survival process activated and maintained in dormant BC cells, which is shut down after the cells undergo the dormant-to-proliferative switch.

### Autophagy inhibition prevents the dormancy-to-growth switch

**Fig. 5:**
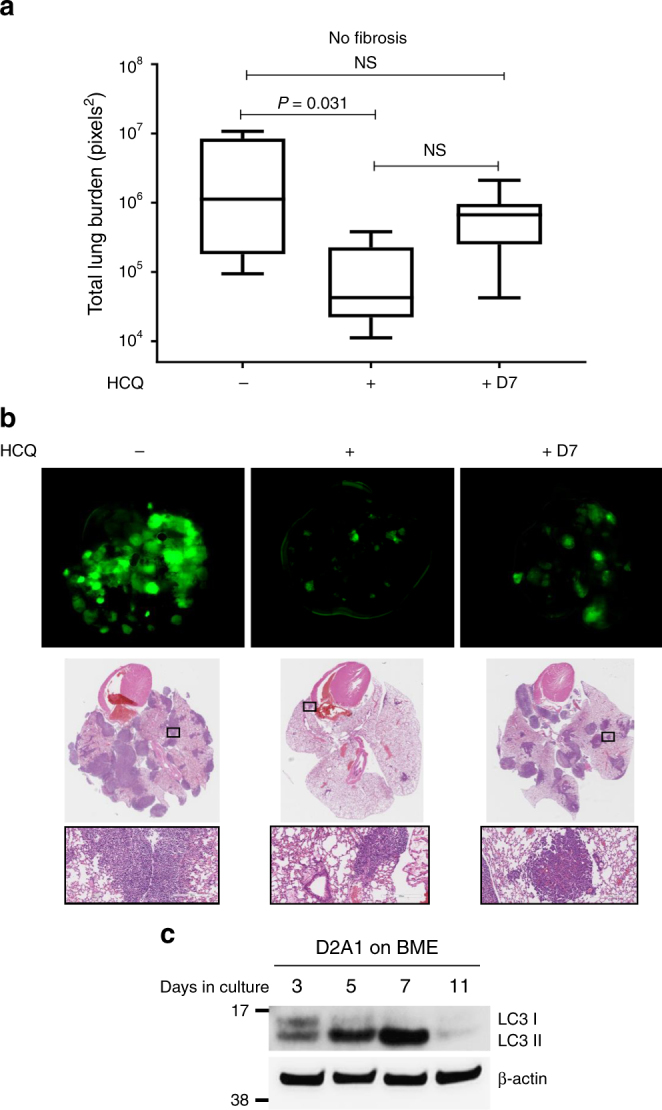
Inhibition of autophagy reduces metastatic outbreak of dormant cells. **a** Total lung surface burden of CD1^nu/nu^ mice receiving Ad-empty (No fibrosis) or Ad-TGF-β^223/225^ (Fibrosis) and tail vein injections of 1 × 10^6^ D2A1-GFP cells. Mice received either vehicle (−), 50 mg/kg body weight of Hydroxychloroquine (HCQ) 5 days a week for 3 weeks (+) or vehicle daily for 7 days followed by 50 mg/kg body weight of HCQ 5 days a week for additional 2 weeks (+D7) (mean ± s.e.m, *n* = 8–9 mice per group. Comparisons by Kruskal–Wallis, Dunn’s post test). **b** Representative images of metastatic lesions in the lung from **a**, upper panels, D2A1 cells expressing GFP; middle panels, H&E staining of cross sections of lungs; bottom panels, higher magnification of H&E stained metastatic lesions. **c** Representative western blot of LC3-I and LC3-II from D2A1 cells plated on BME on days 3, 5, 7 and 11

In contrast to D2.0 R cells, D2A1 exhibit a similar dormant phase in vivo for 1 to 3 weeks after which they form large metastatic lesions^8^. We injected D2A1-GFP cells into the lateral tail vein of  nude mice and performed the same treatment schedule as in Fig. 4a. Average metastatic burden was consistently reduced after 3 weeks of treatment with HCQ as compared to the control and the treatment-delayed groups. In line with previous findings, the delay of HCQ administration for 7 days after cell injection resulted in equivalent metastatic total tumour burden compared to controls (Fig. 5a, b) suggesting that inhibition of autophagy has a minimal effect once the dormant-to-proliferative switch has occurred. To further investigate this effect, we performed a time course experiment addressing the activation status of autophagy in D2A1 cells before and after their proliferative outbreak in vitro. Figure 5c clearly shows the progressive conversion of LC3-I to its lipidated form LC3-II, indicating the activation of autophagy, from day 3 to day 7 of culture. This period of time overlaps with the quiescent phase of D2A1 cells on BME matrices. On the other hand, a sharp decrease in the expression of both LC3 forms is observable at day 11 of culture, at which D2A1 cells have established a stable proliferative profile when cultured on BME matrices.

### Transcriptome analysis of dormant and proliferating BC cells

**Fig. 6:**
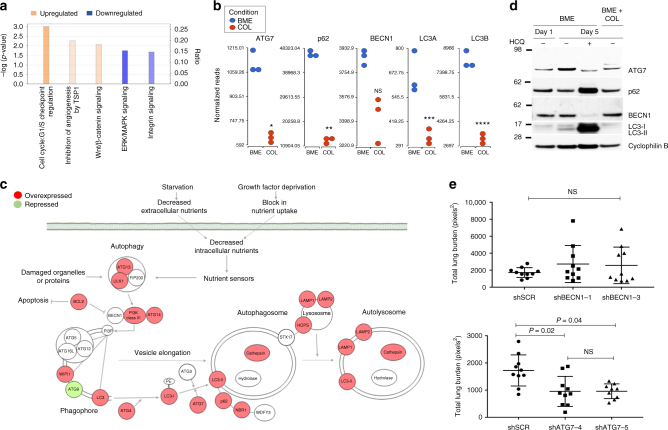
Dormant cells activate a BECN1-independent autophagy pathway. **a** Up and downregulation of breast cancer dormancy-related signalling pathways in D2.0 R cells on BME after 5 days in culture as compared with D2.0 R cells on BME after 1 day in culture. **b** Dot plots representing the differential expression of selected autophagy genes in D2.0 R cells on BME matrices as compared to D2.0 R cells on BME plus COL1 matrices at day 5 of culture (*n* = 3 independent samples per condition. Comparisons by Partek Gene Specific Analysis (GSA) algorithm, false discovery rate (FDR) set at 0.05. *P = 5.45 × 10^−5^; **P = 4.25 × 10^−6^; ***P = 3.05 × 10^−4^; ****P = 9.31 × 10^−6^). **c** Graphical summary of the expression profile of canonical autophagy genes in D2.0 R cells on BME after 5 days in culture as compared with D2.0 R cells on BME plus COL1 after 5 days in culture. **d** Representative western blot of autophagy markers from D2.0 R cells plated on BME or BME plus COL1 with or without HCQ on days 1 and 5. **e** Total lung surface metastatic burden in CD1^nu/nu^ mice injected with D2.0R-GFP stably expressing shSCR (scrambled), shBECN1 (shRNA 1 and 3) (upper panel) or shATG7 (shRNA 4 and 5) (lower panel)

To elucidate the molecular mechanism driving the dependence of dormant BC cells on autophagy, we interrogated the transcriptomes of D2.0 R cells in early and late stages of dormancy and during proliferation. Pathway analysis of differentially expressed genes of D2.0 R at Days 1 and 5 on BME revealed the modulation of signalling pathways previously associated with dormancy, such as the ERK/MAPK pathway, integrin-mediated signalling or angiogenesis inhibition by Thrombospondin 1 (TSP1) (Fig. 6a), but not pathways related to autophagy. We further examined the transcriptomes of D2.0 R cells on BME and COL1-enriched BME matrices at Day 5 of culture. Pathway analysis revealed autophagy as the most prevalent upregulated pathway in the dormant cells compared to proliferating cells at this time point (*p* = 2.5E−11; Fig. 6b, c, Supplementary Table 1 and 2). These results suggest that the onset of the cellular transcriptional programme leading to autophagy occurs relatively early during the transition from a proliferative state in 2D culture to a dormant state on BME.

Given the frequent post-translational modifications associated with autophagy^19^, we validated the transcriptomic data by analysing the protein levels of key molecules in the autophagic signalling pathway. We confirmed that autophagy was active in dormant D2.0 R cells on BME compared to proliferating D2.0 R cells cultured in COL1-enriched BME matrices. We further demonstrated that the protein levels of key autophagy effectors were modulated in accordance with the transcriptional profile of cells seeded on BME at day 5 as compared with those on BME at Day 1 (Fig. 6d). ATG7 and LC3-II were upregulated and p62 downregulated in D2.0 R cells on BME at day 5 as compared with the D2.0 R cells at Day 1, while BECN1 remained unchanged (Fig. 6d). Taken together these data indicate that the differences observed in the autophagic activity of dormant D2.0 R cells reflect changes at the posttranscriptional level.

In addition, we showed that treating dormant D2.0 R cells with HCQ blocked the autophagic flux. Notably, we observed a strong accumulation of p62^22^ and LC3-II^23^ in D2.0 R cells treated with HCQ at day 5 of culture on BME (Fig. 6d). Surprisingly, BECN1 protein levels dramatically decreased upon HCQ treatment.

### Knockdown of ATG7 but not BECN1 reduces metastatic burden

Next, we aimed to confirm the critical role of autophagy in the survival of dormant cells and the involvement of ATG7 and BECN1. We performed an in vivo metastasis burden assay using D2.0R-GFP cells expressing scrambled shRNA (sh-SCR) and two independent short hairpin RNAs (shRNA) for each of the autophagy regulators, BECN1 and ATG7 (Supplementary Fig. 8a). In accordance with the transcriptomic data, we observed no significant differences in the metastatic burden of mice injected with either BECN1 shRNA or sh-SCR cells (Fig. 6e), whereas expression of sh-ATG7 hairpins induced an approximately 2-fold reduction in total lung metastatic burden as compared to controls (Fig. 6f).

### Inhibition of autophagy in dormant cells blocks mitophagy

**Fig. 7:**
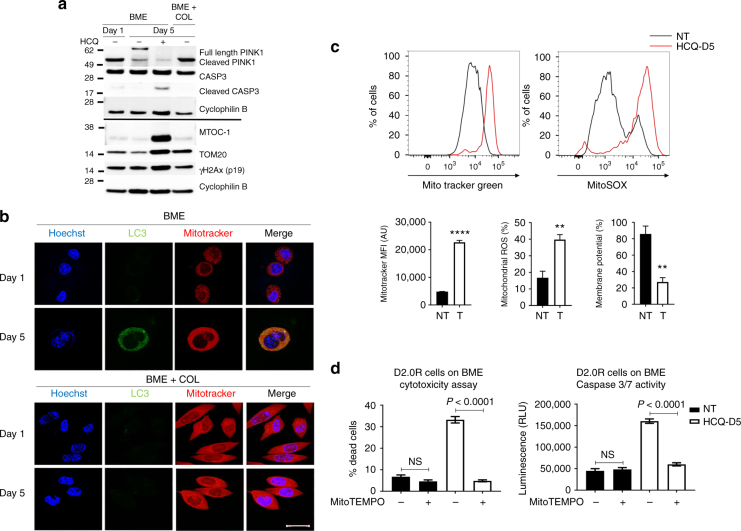
Autophagy inhibition leads to dysfunctional mitochondria and apoptosis. **a** Representative western blot of mitophagy markers from D2.0 R cells plated on BME or BME plus COL1 with or without HCQ on days 1 and 5. **b** Representative images of live D2.0 R cells transfected with the GFP-LC3 reporter and stained with MitoTracker® Red CMXRos on BME or BME plus COL1 matrices for 5 days. Scale bar is 20 µm **c** Mitochondrial (MitoTracker® Green), mitochondrial reactive oxygen species (ROS; MitoSox™) and mitochondrial membrane potential (TMRM) quantification in D2.0 R cells on BME or BME plus COL1 matrices with or without HCQ (mean ± s.e.m, *n* = 60,000 cells from 3 independent experiments. Comparisons by Mann–Whitney *U*-test, two-sided. **P ≤ 0.01; ****P ≤ 0.0001). NT, non-treated; HCQ-D5, hydroxychloroquine treatment beginning on day 5; MFI, mean fluorescence intensity. **d** MitoTempo is protective of ROS-induced cell death in D2.0 R cells on BME treated with HCQ. Cells were pre-treated for 5 days with 20 μM MitoTempo and subsequently exposed to 50 μM HCQ for 24 hrs. Cell viability was assessed by Cytotox Glo assay (left graph. Mean ± s.e.m, *n* = 3 wells. Comparisons by unpaired two-sided *T*-test) and Caspase 3 and 7 activity (right graph. Mean ± s.e.m, *n* = 3 wells. Comparisons by unpaired two-sided *T*-test). Data are representative of three independent experiments

RNAseq data also suggested that genes critically involved in mitophagy and apoptosis were differentially regulated upon autophagy activation (Supplementary Table 2). Mitophagy is a type of selective autophagic survival pathway which eliminates damaged or excessive mitochondria^24^. Western blotting analysis of MTOC-1 and TOM20 showed a striking accumulation of these two mitochondrial markers upon autophagy inhibition in dormant D2.0 R cells on BME at day 5 (Fig. 7a). In addition, we analysed protein levels of PTEN-induced putative kinase protein 1 (PINK1), which activates mitophagy in response to mitochondrial damage^25^. We found that dormant BC cells cultured on BME for 5 days showed an increase in the active 66-kDa full-length PINK1 isoform (Fig. 7a)^26^. A decreased expression of both full length and cleaved PINK1 was observed in the HCQ-treated dormant D2.0 R cells. Consistent with the hypothesis that mitophagy blockade and the accumulation of mitochondria would promote cellular damage and death, we found increased levels of ɣH2Ax and cCASP3 in dormant D2.0 R cells treated with HCQ, as compared with non-treated D2.0 R cells on BME or BME plus COL matrices (Fig. 7a). Considering that dormant D2.0 R cells undergo cell apoptosis following autophagy inhibition, the unexpected decrease in the expression of BECN1 and PINK1 in dormant D2.0 R treated with HCQ (Figs. 6d and 7a, respectively) might reflect the increased susceptibility of these molecules to proteolysis during apoptosis. Indeed, caspase-mediated cleavage of BECN1 has been previously reported^27^. Indeed, we observed a sharp decrease in the expression of other cytoplasmic proteins upon HCQ treatment of D2.0 R cells on BME, such as β-actin or α/β-tubulin (Supplementary Fig. 8b), possibly accounting for the high proteolytic activity in apoptotic D2.0 R cells under these conditions.

Analysis of the potential physical interaction between autophagosomes and mitochondria in dormant D2.0 R cells revealed that an average of 75% of the mitochondrial mass in dormant D2.0 R cells on BME co-localised with LC3-GFP in autophagosomal structures at day 5 of culture, while no co-localisation was detectable in proliferating cells on BME plus COL1 at any time (Fig. 7b). Consistent with these findings, dormant D2.0 R cells on BME at day 5 accumulated more mitochondria when treated with HCQ compared to untreated D2.0 R cells. Furthermore, the accumulation of mitochondria in dormant D2.0 R cells treated with HCQ was accompanied by an increase in mitochondrial ROS and a decrease in mitochondrial membrane potential (Fig. 7c), suggesting that the blockade of autophagic flux inhibited the clearance of damaged mitochondria producing toxic by-products, including mitochondrial superoxide. The later observation was confirmed by rescuing D2.0 R viability upon treatment with HCQ through the addition of the mitochondrial-targeted ROS scavenger MitoTEMPO to the 3D cultures. Both the percentage of dead D2.0 R cells and the caspase 3 and 7 activities were significantly decreased by the presence of MitoTEMPO in the 3D culture after cells were treated with HCQ at day 5 of culture (Fig. 7d).

## Discussion

The role of autophagy in cancer is complex since it can both suppress cancer initiation and promote the growth and maintenance of established tumours^28^. Recent investigations suggest that autophagy facilitates multiple steps in the metastatic cascade^29,30^. Indeed, a role for autophagy in supporting tumour cell dormancy has been suggested^15^ but experimental evidence has been limited^17,18^. The results described in this study demonstrate that BC cells utilise autophagy while in a dormant state. Further, inhibition of autophagy drastically impairs the survival of dormant BC cells in vitro and in vivo.

Importantly, autophagy inhibition does not induce apoptosis in proliferating cells, underscoring the specificity of the toxic effect of autophagy blockade in dormant BC cells. Although autophagy inhibition impedes the survival of dormant D2.0 R BC cells in 3D culture and in vivo, delaying the anti-autophagic treatment until after the dormant-to-proliferative switch has occurred, significantly reduces the cell population undergoing apoptotic cell death. Importantly, extended follow-up of the animals after treatment with HCQ confirmed the therapeutic efficacy of autophagy inhibition against dormant DTCs, since we repeatedly observed a significant decrease of the metastatic tumour burden in mice treated with HCQ following tumour cell injection compared to controls. However, when treatment with HCQ was delayed for 7 days following cell injections, we no longer observed significant differences in the metastatic tumour burden of untreated vs treated mice bearing fibrotic lungs. These data further confirmed the insensitivity of proliferating DTCs to autophagy inhibition.

These results were not limited to D2.0 R cells. We observed that after a short latency period during which the highly metastatic D2A1 and MDA-MB-231 cell lines were sensitive to HCQ treatment, the cells were protected from the induction of apoptotic cell death upon treatment with autophagy inhibitors once they began to spontaneously proliferate. These observations were confirmed in vivo through the demonstration that D2A1 cells were insensitive to autophagy inhibition once they had undergone the spontaneous dormant-to-proliferative switch. This was in sharp contrast with the prominent decrease in metastatic tumour burden observed in mice treated immediately with HCQ following D2A1 cell injection. Notably, the two cell lines exhibiting a stable dormant phenotype in our model system, D2.0 R and MCF7^8^, were sensitive to autophagy inhibition regardless of when autophagy inhibition was initiated.

The dependence of dormant BC cells on autophagy was further demonstrated through examination of the autophagic flux in D2.0 R cells exposed to both non-fibrotic (dormant) and fibrotic (proliferative) lung environments. We have demonstrated that autophagy is a critical process during the early phase of metastatic dissemination of BC cells in both growth restrictive and growth permissive microenvironments. However, proliferating BC cells are significantly less sensitive to autophagy inhibition compared to their dormant counterparts. These data further demonstrate that disseminated BC cells undergo an initial quiescent phase in the metastatic colonisation process, which is highly dependent on autophagy. The duration of the quiescent phase might be variable depending on multiple factors, among them the metastatic capacity of the cells and microenvironmental conditions^31^.

In addition, our results suggest that as quiescent BC cells transition into a proliferative phase, autophagy is downregulated and no longer appears critical for their maintenance as other survival signals likely become activated. This observation might provide an explanation for the discordant results from clinical trials addressing the clinical efficacy of autophagy inhibitors in cancer treatment. Our data suggests that inhibition of autophagy may primarily affect survival of dormant/quiescent cells prior to their adaptation to a new metastatic microenvironment. Once established in their new environment or after proliferation has been initiated, cells no longer require autophagy and therefore would not be expected to respond to autophagy inhibition, resulting in the lack of response in patients diagnosed with either early stage disease or clinically detectable metastasis. It is also possible that cells in the primary tumour or proliferating in overt metastasis use other survival pathways besides autophagy or following an initial phase of autophagy in which case autophagy inhibition may not be effective.

A limitation of the model systems used in these studies is that the induction of the dormant-to-proliferative switch by collagen or fibrosis is not reversible and that once this occurs, autophagy is no longer a critical survival pathway. It will be of interest in the future as more sophisticated dormancy models are developed to determine whether the autophagy survival pathway is reactivated if proliferating cells can be induced to enter dormancy. Also, metastatic disease is the result of the interaction of a myriad of factors, both tumour autonomous and tumour non-autonomous. The tumour non-autonomous factors include collagen^32^, a property exploited by our in vitro and in vivo model systems^8–10^, but also many other molecules and cellular interactions which contribute to the metastatic process^2^.

Our data indicate that transcriptional expression of the autophagy pathway is an early event in dormancy. Interestingly, we also found that BECN1 expression was unchanged across the entire dataset as well as the protein level. Although knocking down the expression of BECN1, a key regulator of autophagy, in D2.0 R cells did not affect the average metastatic burden in vivo, knockdown of ATG7 significantly reduced the metastatic capacity of D2.0 R cells as compared to control cells. These data indicate that autophagy occurs in dormant D2.0 R cell following a non-canonical signalling pathway, which is independent of BECN1.

Transcriptomic data of dormant BC cells also revealed the activation of mitophagy, a selective form of autophagy corresponding to the engulfment and degradation of damaged or excessive mitochondria^24^. We observed a striking accumulation of mitochondrial proteins upon autophagy inhibition, further suggesting that mitochondria constituted a significant proportion of the autophagosomal cargo in BC dormant cells. There are three major pathways involved in mitophagy activation, including the pathway activated by PINK1 stabilisation in the mitochondrial outer membrane^33^. Indeed, we found that dormant BC cells exhibited an accumulation of the active form of PINK1 at day 5 of culture on BME matrices. In addition, we have reported a high degree of co-localisation between LC3 and mitochondrial markers in dormant BC cells together with a decrease in mitochondrial membrane potential and an increase in mitochondrial ROS and mitochondrial mass in dormant BC cells treated with HCQ as compared with their non-treated counterparts. It is well known that impaired mitochondria produce a high level of ROS and consequently, cellular damage^34,35^. Increased levels of ɣH2Ax and cCASP3 upon autophagy inhibition in dormant BC cells further highlighted the relevance of mitochondrial quality control in the maintenance of quiescent BC cell integrity.

The data from this study provide compelling evidence for the vital role of autophagy in BC dormancy, in which the clearance of damaged mitochondria and maintenance of redox homoeostasis in the early stages of the metastatic colonisation appear to play an important role. These results suggest that the inclusion of therapeutic approaches to inhibit autophagy as part of the treatment of primary BC tumours or as an adjuvant therapy might reduce the dormant tumour cell population responsible for BC recurrence and ultimately improve the survival rates of BC patients.

## Methods

### Cell lines and cell culture

Mouse mammary tumour D2.0 R and D2A1 cells were derived from spontaneous hyperplastic alveolar nodules^13,36^ and provided by Ann Chambers (London Cancer Center, London, Ontario, Canada). MDA-MB-231 and MCF7 cells were obtained from American Type Culture Collection (ATCC, HTB-26 and HTB-22, respectively). The cells were grown in 2D and 3D cultures^8^. Briefly, cells grown in 2D were maintained in DMEM high glucose (4.5 g/L glucose, Gibco, 11965), 10% fetal bovine serum (FBS) at 37 °C and 5% CO_2_. To generate the 3D in vitro systems, the cells were cultured in growth factor–reduced three-dimensional Cultrex® Basement Membrane Extract (BME) (obtained from Trevigen, Gaithersburg, MDUSA;3432-005-01). Cell culture plates and chamber slides were coated with 150 μl BME or BME and COL1 per cm^2^. 1.5 × 10^4^ cells/ml were resuspended in DMEM low glucose (1 g/L glucose, Gibco, 11885) either supplemented with 2% FBS and 2% BME or 2% FBS and BME plus COL1 (2 mg/ml final COL1 concentration). Cells were cultured on the coated slides at 37 °C and 5% CO_2_ and re-fed every 5 days. Cultrex® Basement Membrane Extract (BME) (obtained from Trevigen, Gaithersburg, MDUSA;3432-005-01), is extracted from Engelbreth-Holm-Swarm (EHS) tumours as a solubilized basement membrane. The extract primarily contains laminin, collagen IV, entactin, and heparan sulphate proteoglycan. The BME used in these experiments has been processed to minimise matrix-associated growth factors. It will polymerise to form a basement membrane matrix hydrogel at 37 °C which provides an extracellular matrix environment for the 3D in vitro culture conditions used in these studies (https://trevigen.com/product-category/physiologic-cell-culture/extracellular-matrix-proteins/). D2.0 R and D2A1 stably expressing GFP were generated via lentiviral infection of the pSICO construct (provided by Tyler Jacks, Massachusetts Institute of Technology, Cambridge, Massachusetts, USA). HCQ, 3-MA and Bafilomycin were used as autophagy inhibitors (Sigma-Aldrich). MitoTEMPO was used as a mitochondrial ROS quencher at 20 µM in vitro.

### Expression of mCherry-EGFP- LC3 and GFP-LC3 in D2.0 R cells

pBABE retroviral vectors containing mCherry-EGFP- LC3 (Addgene, 22418) or GFP-LC3 (Addgene, 22405) were transfected into PT67 cells (ATCC) using Lipofectamine 3000 (Life Technologies). Retrovirus-containing suspension was collected after 48 h, passed through a 45um filter to obtain viral particles and D2.0 R were transduced in suspension while spinning at 180×*g* for 1 h and subsequently selected with puromycin.

### Knockdown of ATG7 and BECN1

TRC lentiviral shRNA constructs targeting ATG7 (RMM4534-EG74244; Clone IDs: TRCN0000092163, TRCN0000092164, TRCN0000092165, TRCN0000092166 and TRCN0000092167), BECN1 (RMM4534-EG56208; Clone IDs: TRCN0000087288, TRCN0000087289, TRCN0000087290, TRCN0000087291 and TRCN0000087292) or scrambled shRNA (RHS6848) were purchased from Dharmacon. 293 T cells were transfected with 1ug of shRNA and 1ug of viral packaging plasmids (250ng pMD2.G and 750 ng psPAX2) using 6µl of Xtreme Gene 9 transfection reagent (Roche). After 48 h, virus-containing supernatant was passed through a 45 µm filter to obtain viral particles, which were then transferred to 50,000 D2.0 R cells infected in suspension while spinning at 180×*g* for 1 h. Finally, 48 h after transduction, the cells were selected with puromycin and the knockdown verified by western blot.

### Cytotoxicity assay

1.5 × 10^3^ cells were resuspended in 100 μl DMEM (1 g/l glucose; Invitrogen) supplemented with 2% FBS and 2% Cultrex basement membrane extract (BME) (Trevigen) or 2% FBS and BME plus COL1 (2 mg/ml final COL concentration), with or without HCQ, and were grown in triplicate in 96-well plates coated with 50 μl BME or BME plus COL1. The % of dead cells were measured using the Cytotox-Glo Cytoxocity assay (Promega), according to the manufacturer’s instructions. Briefly, 25 µl of cytotox-Glo reagent was added to each well, incubated for 10 min and luminescence was read to assess cytotoxicity. For the assessment of total cell number, 25 µl of lysis reagent was added followed by 10 min incubation and luminescence read in a Glomax (Promega) microplate luminometer.

### Caspase 3/7 activity assay

Caspase 3/7 activity was performed using the Caspase-Glo 3/7 Assay (Promega) according to the manufacturer’s protocol. Cells were seeded in 3D cultures as described above with or without 20 μM MitoTEMPO for 5 days. Cells were treated with HCQ at day 5 and assayed after 24 h. Briefly, plates and reconstituted caspase 3/7 reagent were allowed to equilibrate to room temperature (RT), reagent was then added to wells at a 1:1 ratio and incubated for 1 h at RT. Luminescence was measured using aGlomax (Promega) microplate luminometer. Wells with all assay components, except the cells, served as blank controls.

### Proliferation assay

Cells were plated in 3D cultures as described above with or without HCQ and were grown in triplicate in 96-well plates coated with 50 μl BME or BME plus COL1. Proliferation was measured using the CellTiter 96® AQueous One Solution Cell Proliferation Assay (Promega), respectively, according to the manufacturer’s instructions. Briefly, 20 µl of CellTiter 96® AQueous One Solution were added to each well, and the absorbance at 490 nm was recorded 2 h later.

### BrdU incorporation assay

Proliferation was analysed using the BrdU Cell Proliferation Assay Kit (Cell Signaling Technology,6813) according to the manufacturer’s instructions. Cells were pulsed with BrdU for 1 h.

### Western blot analysis

Protein was extracted from 3D cultures as previously described^37^. Western blotting was conducted as previously reported^9^, with minor modifications. Briefly, 10 μg of total protein were loaded in NuPAGE™ 4–12% Bis–Tris Protein Gels (Invitrogen). After separation, proteins were transferred to PVDF membrane (Millipore). Primary antibodies used were: from Cell Signaling Technology anti-ATG7 (8558, dilution 1/1000), p62 (5114, dilution 1/1000), BECN1 (3495, dilution 1/1000), LC3 (12741, dilution 1/1000), CASP3 (9662, dilution 1/1000), cleaved CASP3 (9661, dilution 1/1000), ɣH2Ax (p139S) (2577, dilution 1/1000), from Abcam anti-PINK1 (ab75487, dilution 1/500), MTOC-1 (ab14705, dilution 1/2000), TOM20 (ab56783, dilution 1/500) and from Thermo Scientific anti-Cyclophillin B (PA1-027A, dilution 1/300), followed by secondary antibodies (GE Healthcare). Blots were imaged in a ChemiDoc™ Imaging Systems (Biorad). Uncropped images of western blots are shown in Supplementary Figures 9 and 10.

### Immunofluorescence

Cells were plated in 3D cultures as described above in 24-well plates with or without HCQ. Well coatings containing the cells were smeared on permafrost slides and fixed for 10 min with 4% PFA. The cells were washed 10 min with PBS and permeabilized with PBS containing 0.5%Triton X-100 (Sigma) for 10 min, at 4 °C. Permeabilized cells were washed with PBS (3 × 15 min) and blocked with 10% goat serum for 1 h and incubated overnight at 4 °C with primary antibody. Slides were incubated with anti-LAMP1 from Novus Biologicals (NBP2-25154) and from Cell Signaling Technology anti-LC3 (CST, 12741), Ki67 (CST 9449) or cleaved CASP3 (CST, 9661) in 5% BSA and Tris-buffered saline and 0.05% Tween 20 (TBS-T) at 4 °C overnight. Later, the slides were incubated with either Alexa fluor 488 goat anti-rabbit and Alexa fluor 568 goat anti-mouse secondary antibodies or Alexa fluor 488 goat anti-mouse and Alexa fluor 594 goat anti-rabbit (Invitrogen) for 1 h, mounted with Vectashield mounting medium with 4’,6-diamidino-2-phenylindole (DAPI) and imaged using a Zeiss LSM 780 confocal microscope.

### Live cell imaging

Cells were plated in 3D cultures as described above in µ-slides (Ibidi) with or without HCQ. Prior to the observation of the cultures, the cells were incubated with 500 nM MitoTracker Red CMXRos (M7512, Invitrogen) and/or Hoechst 33342 (R37605, Invitrogen) and subsequently imaged using a Zeiss LSM 780 confocal microscope.

### Digital image acquisition and processing

Digital images were acquired using: (1) an inverted microscope EVOS FL auto (Thermo Fisher Scientific); (2) confocal images of 3D cultures were taken using Zeiss LSM-780 confocal system for which acquisition was performed using Zeiss LSM software ZEN. Images were composed and edited in ZEN or Photoshop CC (Adobe) software, in which background was reduced using brightness and contrast adjustments applied to the whole image. Co-localisation of GFP-LC3 and MitoTracker RedCMXRos was determined using a ZEN automated macro pipeline calculating double-positive pixels.

### Flow cytometry analysis

To isolate mCherry-GFP-LC3 D2.0 R cells, the lungs were mechanically disaggregated (gentleMACS, Miltenyl Biotec) and filtered through 40 µm and 10 µm cell strainers. Cells isolated from BME matrices^37^ were stained using 100 nM MitoTracker Green FM (M7514), mitochondrial ROS using 5 μM MitoSox (M36008) and mitochondrial membrane potential using 250 nM TMRM (T-668), following the manufacturer’s protocol (Invitrogen). Prior to FACS, cells were stained with Pacific blue (Invitrogen) to discriminate live and dead cells and directly analysed without fixing. Data acquisition of the stained cells was performed on LSR–Fortessa cytometer and the analysis was performed on FlowJo software. Autophagic flux was determined by plotting the ratio of mCherry:EGFP in D2.0 R stably expressing the tandem reporter mCherry-GFP-LC3. To define low and high autophagic flux cell populations we performed a preliminary study in which we subjected D2.0 R mCherry-GFP-LC3 cells to starvation conditions (cells cultured in or DMEM 2 g/dL glucose + 2% FBS) while in culture for 6 days. We used these cells as an autophagy positive control and compared the resulting mCherry:EGFP ratio to that obtained from D2.0 R mCherry-GFP-LC3 cells grown in complete media (DMEM 4.5 g/dL glucose + 10% FBS) for 6 days. Starved D2.0 R mCherry-GFP-LC3 cells showed a higher mCherry:EGFP ratio as compared to non-starved D2.0 R mCherry-GFP-LC3 cells (Supplementary Fig. 7c). These cell populations were defined as high autophagic flux and low autophagic flux D2.0 R mCherry-GFP-LC3 cells and the analysis settings used in this experiment were later applied to analyse the flow cytometry data from D2.0 R mCherry-GFP-LC3 cells isolated from mouse lungs in the experiment reported in Fig. 4f of the main text.

Mean fluorescence intensity (MFI) values were normalised to the mitochondrial mass of the cells and calculated to determine the percentage of mitochondrial ROS and the percentage of mitochondrial membrane potential. To do that, we calculated the MFI ratio of Mitosox or TMRM to Mitotracker Green in order to obtain normalised MFI values for mitochondrial ROS and mitochondrial membrane potential, respectively. Values were expressed as a percentage of positively stained cells out of the entire cell population analysed.

### RNAseq and bioinformatics analysis

Cells were isolated from the 3D matrices^37^. Briefly, culture matrices were incubated with 2 ml of ice cold DPBS, 5 mM EDTA solution at 4 °C in agitation for 60 min. Solubilized culture matrices and cells were transferred to a tube and spun at 3200 xg, 4 °C for 10 min. Supernatant was discarded and pellets resuspended in 10 ml of ice cold DPBS, 5 mM EDTA solution. Resuspended cells were spun at 1700×*g* 4 °C for 15 min. This step was repeated twice or until the cell pellets were visible. Following cell extraction from the matrices, RNA was extracted using the RNeasy kit (Qiagen) following the manufacturer’s instructions. The quality and quantity of extracted total RNA was assessed using TapeStation 2200 (Agilent). All RNA sequencing (RNA-seq) analyses were performed using three biological replicates. Library preparation was performed using the NEBNext Ultra Directional RNA Library Prep Kit for Illumina with NEBNext multiplexing oligos using the manufacturer’s protocol. RNA sequencing was conducted on the Illumina HiSeq 2500. Aligned reads (BAM files) were analysed using PartekFlow software for differential expression and gene enrichment analysis. Comparisons used Partek Gene Specific Analysis (GSA) algorithm and multiple comparisons were corrected using false discovery rate (FDR), which was set at 0.05. Ingenuity Pathway Analysis was applied to differentially expressed gene sets to identify the pathways that changed across the dataset.

### Animal studies

Study approval: All animals were treated in accordance with the guidelines of the Animal Care and Use of Laboratory animals (NIH publication no. 86–23, 1985) under the protocol LCBG-033 approved by the IACUC of the National Cancer Institute (NCI).

Pulmonary fibrosis: Pulmonary fibrosis was induced in 8-week-old female CD1^nu/nu^ athymic mice (Charles River Laboratories)^38^. Mice received 5 × 10^8^ PFU of adenoviral vector expressing either active TGFβ1 (Ad-TGFβ1223/225) to induce fibrosis or adenovirus-null vector control (Ad-empty) in 20 μl PBS to the lungs through nasal instillation.

Experimental metastasis assay: 21 days after infection, 1 × 10^6^ D2.0R-GFP cells were tail vein injected into CD1^nu/nu^ athymic mice. Alternatively, 1 × 10^6^ sh-SCR, sh-BECN1 (shRNA 1 and 3) and sh-ATG7 (shRNA 4 and 5) D2.0R-GFP cells, mCherry-EGFP-LC3 D2.0 R cells or D2A1-GFP cells were tail vein injected into CD1^nu/nu^ athymic mice that did not receive adenovirus.

In vivo treatment: Autophagy was blocked in CD^nu/nu^ athymic mice by injecting 50 mg per Kg of body weight of HCQ or vehicle (PBS) i.p. 5 days a week for 3 weeks.

Lung imaging: At the experimental endpoint, mice were killed, their lungs extracted, perfused with PBS and imaged by fluorescent single cell organ microscopy (SCOM) imaging (EVOS FL Auto, Thermo Fisher Scientific)^9^. Images of the total surface of the lungs were captured at ×10 magnification and analysed using ImageJ software (NIH).

### Statistics

In vitro experiments were repeated at least 3 times with 3 technical replicates each with representative images, blots and histograms shown. All data are represented as the mean ± standard error of the mean (SEM; represented as error bars in the graphs). Shapiro-Wilks test was used to assess data normality and, according to the results, a parametric or a non-parametric test was chosen, as specified in the Fig. legends. *P* ≤ 0.05 was considered significant. Details of the statistical analyses of metastatic lesion measurements by SCOM^9^ are as follows. Pixel counts of GFP-positive metastatic lung lesions were quantified for the entire surface area of each lung. Lesions with >10 and <1000 pixels^2^ represented individual tumour cells; lesions with >1000 pixels^2^ represented multicellular metastatic lesions. Total tumour burden/lung was represented by the total number of pixels^2^ detected on the entire surface of each lung. Average tumour burden/lung was represented as the mean size of the metastatic lesions. Statistical analyses of differences in the distribution of tumour burden between groups utilised Mann–Whitney *U*-test for pairwise comparisons and Kruskal–Wallis test for multiple group comparisons with Dunn’s correction. Statistical significance was set at *P* < 0.05.

### Data availability

Sequencing data that support the findings of this study have been deposited in the National Center for Biotechnology Information Gene Expression Omnibus (GEO) and are accessible through the GEO Series accession number GSE112094. All other relevant data are available from the corresponding author on request.

## Electronic supplementary material

Supplementary Information
