# Supplementary material for: Autophagy promotes the survival of dormant breast cancer cells and metastatic tumour recurrence
Source: Nat Commun. 2018 May 22;9:1944. doi: 10.1038/s41467-018-04070-6 (PMC5964069; doi:10.1038/s41467-018-04070-6)
Supplement: Supplementary file 1 — Supplementary Information [file 41467_2018_4070_MOESM1_ESM.pdf]

Supplementary Information

Autophagy promotes the survival of dormant breast cancer cells and metastatic tumour recurrence.

Vera-Ramirez et al.

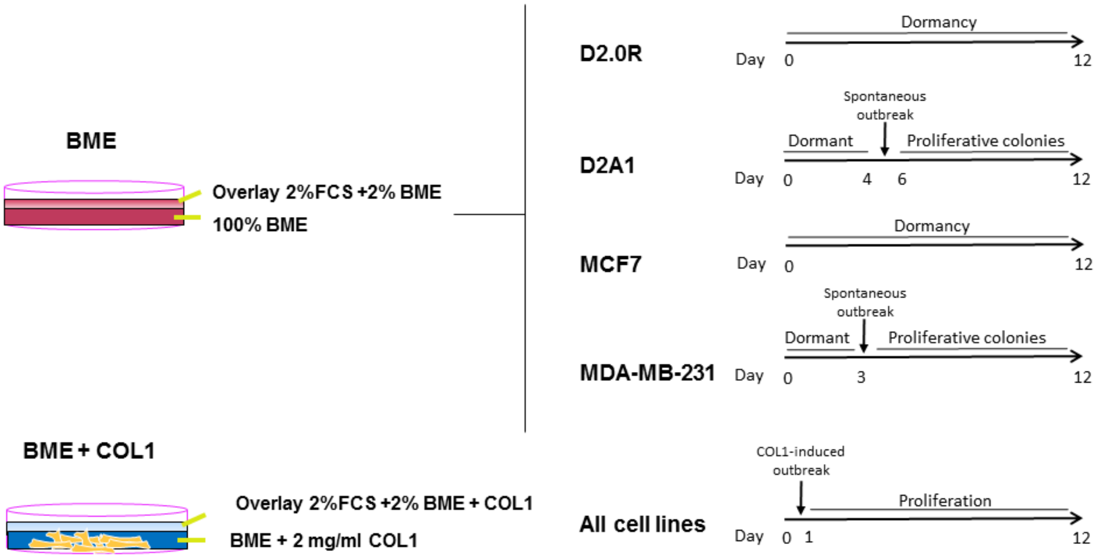

Supplementary Fig. 1. *In vitro* growth kinetics in 3D cultures of mouse and human breast cancer cell lines. Schematic representation of the dormant/proliferative behaviour of each cell line in 3D culture. The proliferative behaviour of the cells on BME alone are shown at the top of the figure, while the phenotype on BME + COL1 is shown at the bottom.

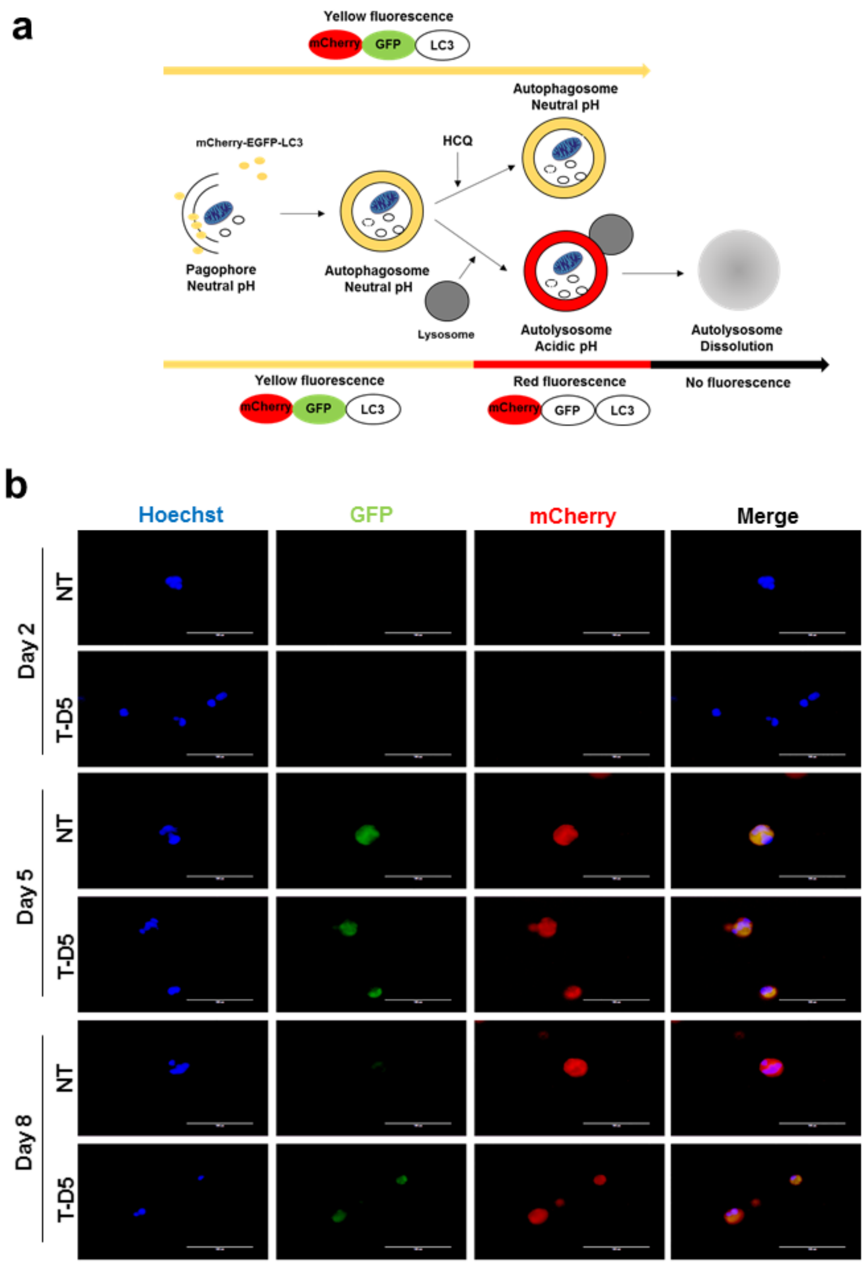

Supplementary Fig. 2. Tracking autophagy with double-tagged LC3B. **a.** Schematic representation of the tandem fusion of mCherry and GFP tagged LC3B pH-sensitive biosensor that is used to monitor autophagy in live cells. The GFP tag is acid-sensitive

while the mCherry tag is acid-insensitive. Therefore, the double-tagged LC3 protein (mCherry-GFP-LC3) will emit yellow (green merged with red) fluorescence in non-acidic structures (autophagosomes) whereas it appears as red only after lysosome-autophagosome fusion (autolysosomes) due to quenching of GFP in these acidic structures. When the fusion between lysosome and autophagosome is blocked [i.e. by treating the cells with lysosomotrophic agents, such as hydroxychloroquine (HCQ)] the switch of yellow to red fluorescence is not observed. **b.** Representative images of live D2.0R cells transfected with the mCherry-EGFP-LC3 reporter, grown on BME for 2, 5 and 8 days. Cultures were treated with vehicle (NT) or HCQ at day 5 of culture (T-D5). Cells show the transition from non-acidic autophagosomes (yellow fluorescence) at day 5 of culture to acidic autolysosomes (red fluorescence) at day 8 of culture. Note that when treated with HCQ, D2.0R mCherry-GFP-LC3 cells do not transition to red only fluorescence at day 8 of culture. Scale bar is 100µm. HCQ, hydroxychloroquine; D5, cells treated with HCQ after 5 days in culture

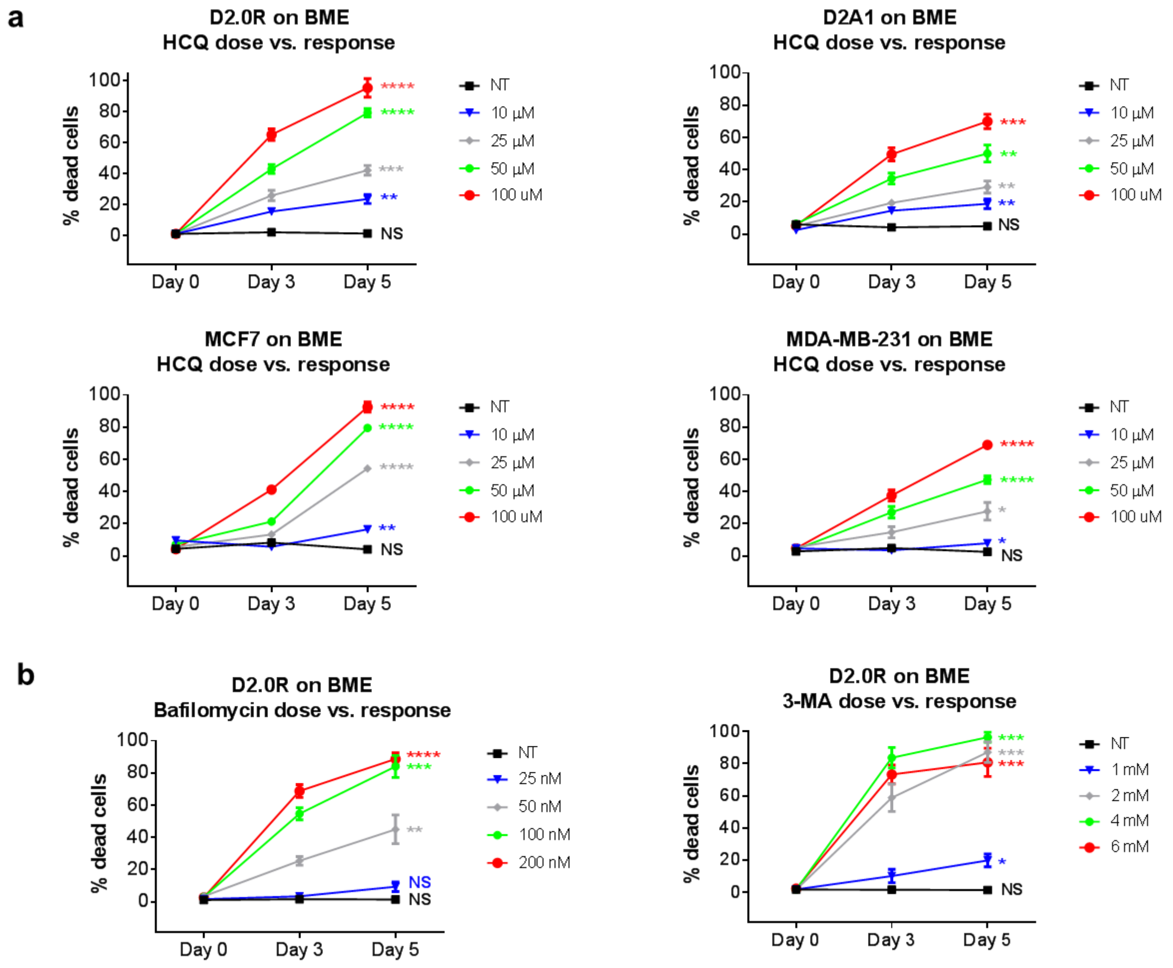

Supplementary Fig. 3. Autophagy inhibition through the use of different drugs promotes cell death in several breast cancer cell lines on BME matrices. a. Hydroxycholoroquine (HCQ) induced a dose-dependent reduction in the proportion of viable D2.0R, D2A1, MCF7 and MDA-MB-231 cells on BME, as determined by cytotoxicity assay (mean ± s.e.m, n=3 wells. Comparisons by unpaired two-sided T test. **P*≤0.05, ***P*≤0.01, ****P*≤0.001 and *****P*≤0.0001). b. 3-methylalanine (3-MA) and Bafilomycin induced a dose-dependent reduction in the proportion of viable D2.0R cells on BME, as determined by cytotoxicity assay (mean ± s.e.m, n=3 wells. Comparisons by unpaired two-sided T test. **P*≤0.05, ***P*≤0.01, ****P*≤0.001 and *****P*≤0.0001).

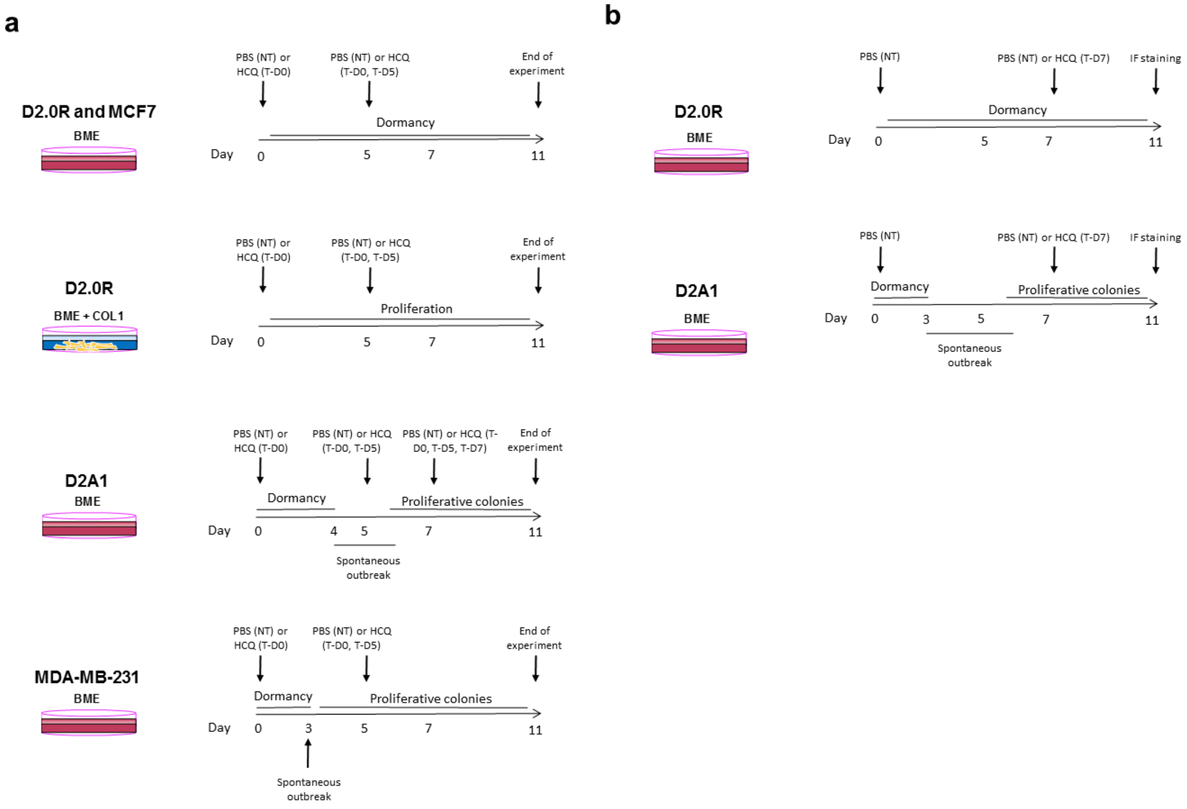

Supplementary Fig. 4. Experimental design of *in vitro* cytotoxicity and proliferation assays using 3D cultures. a. Design of the cytotoxicity and proliferation assays. Note that the dormant/proliferative state of the cell lines at the time that treatment was administered is indicated along the experimental timeline. Only D2.0R cells were plated on BME plus COL1 **b.** Design of the immunofluorescence staining. Treatment with HCQ was delayed until day 7 of culture to allow the spontaneous outbreak of D2A1 cells and keep a consistent treatment schedule across all cell lines used in the experiment. NT, non-treated; HCQ, hydroxychloroquine; D0, cells treated with HCQ immediately after plating on Day 0 and repeated at Day 5; D5, cells treated with HCQ after 5 days in culture; D7, cells treated with HCQ after 7 days in culture.

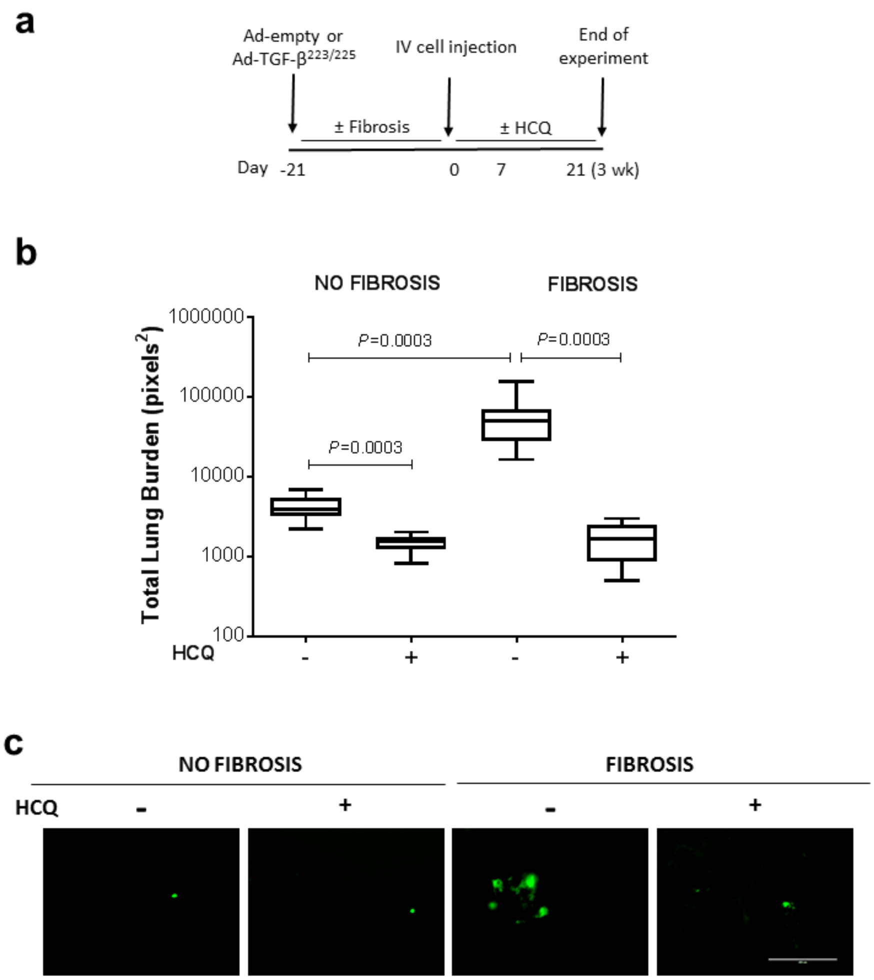

Supplementary Fig. 5. Autophagy inhibition reduces the lung tumor burden of D2.0R cells in both non-fibrotic lungs favoring dormancy and fibrotic lungs inducing metastatic proliferation. a. Experimental design **b.** Total lung surface tumor cell burden of CD1$^{nu/nu}$ mice receiving Ad-empty (no fibrosis) or Ad–TGF-β$^{223/225}$ (fibrosis) and tail-vein injections of 1×10^6 D2.0R GFP cells, followed by vehicle (-) or 50 mg/kg body weight of Hydroxychloroquine (HCQ) 5 days a week for 3 weeks (+) (mean ± s.e.m, n=9-11 mice per group. Comparison by Mann-Whitney U-test, two-sided, Bonferroni post-test). c. Representative images from the experiment in b of single D20R dormant cells in

lungs without fibrosis (left panels) and clusters of D20R cells in metastatic lesions in fibrotic lungs (right panels) in untreated mice. Note that multicellular metastatic lesions were not visible in mice with fibrotic lungs treated with HCQ seven days after cell injection (right panel). Scale bar is 400 μm.

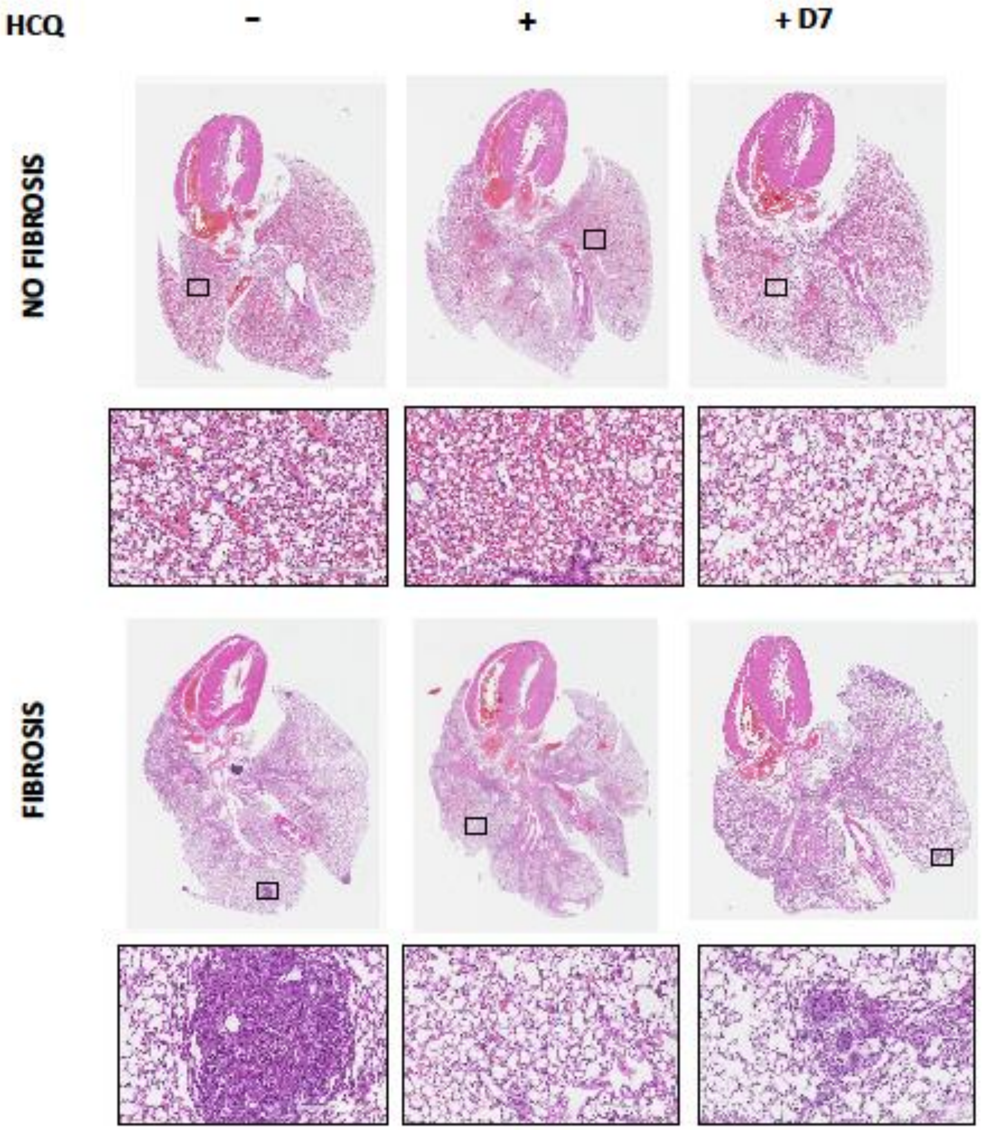

Supplementary Fig. 6. H&E staining of mouse lung sections from the *in vivo* experiment shown in Figure 4b and c. Representative images of single dormant cells and metastatic lesions in the lung from the experiment in Fig. 4b. Note that metastatic lesions are only observable and presented as high magnification images in mice with fibrotic lungs either treated with vehicle or treated seven days after cell injection. Scale bar is 300 µm for high magnification images.

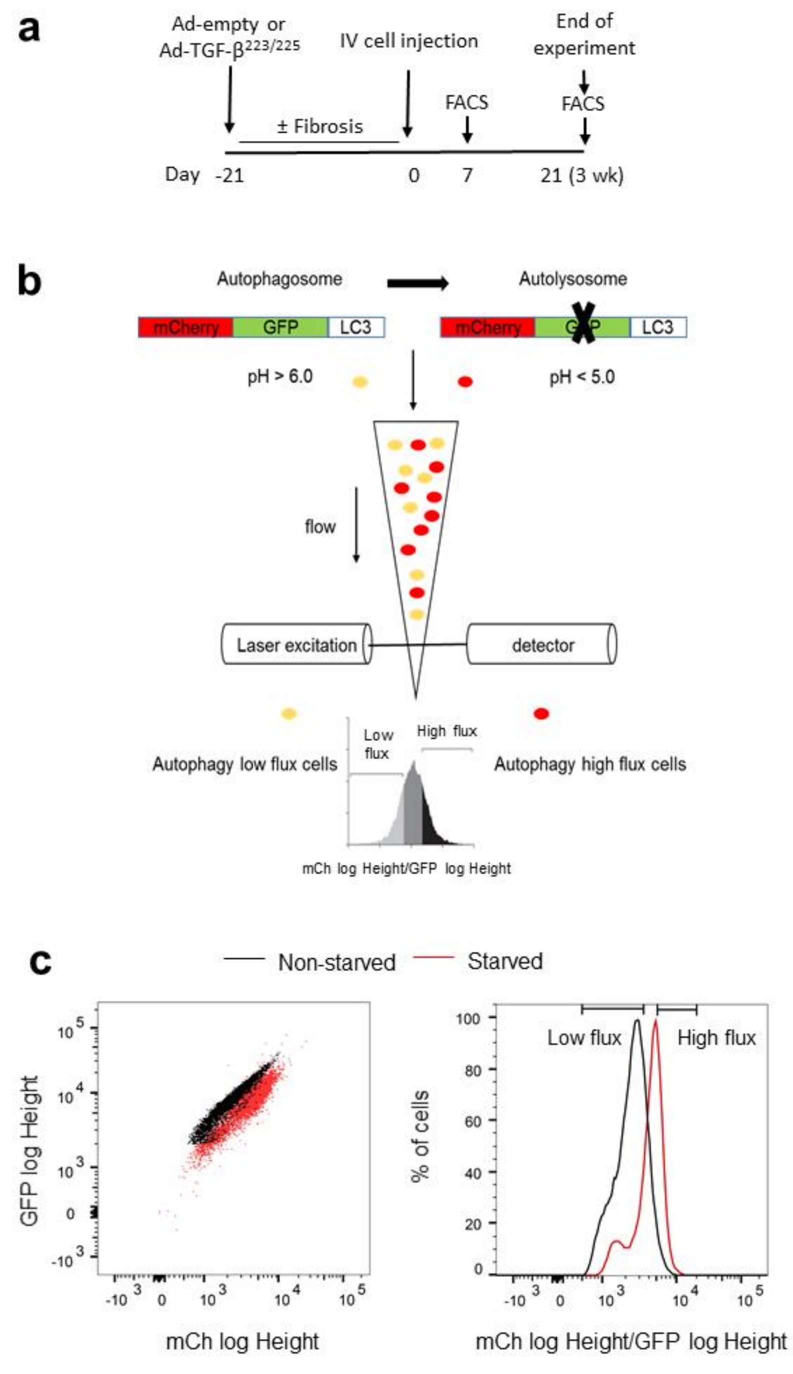

Supplementary Fig. 7. Establishment of experimental settings to analyze autophagic flow *in vivo*. a. Outline of the FACS experiment for the results shown in Figure 5f. **b.** Scheme of the strategy followed to perform the FACS analysis of D2.0R

mCherrry-EGFP-LC3 cells isolated from the lungs of $CD1^{nu/nu}$ mice receiving Ad-empty (No fibrosis) or Ad–TGF-$\beta^{223/225}$ (Fibrosis). Cells constitutively expressing mCherry-GFP-LC3 were analyzed based on the relative ratio of mCherry/EGFP fluorescence which changes in response to the pH that switches from neutral to acidic as autophagosomes fuse with lysosomes to form autolysosomes. **c.** To determine the threshold value separating high from low autophagic flux cells (see Supplementary Methods for details), D20R mCherry-EGFP-LC3 cells were grown in complete DMEM (4.5 g/dL glucose) + 10% FBS (non-starved, low autophagic flux) or low-glucose (2 g/dL glucose) DMEM and 2% FBS (starved, high autophagic flux) for 6 days followed by flow cytometry analysis for autophagic flux.

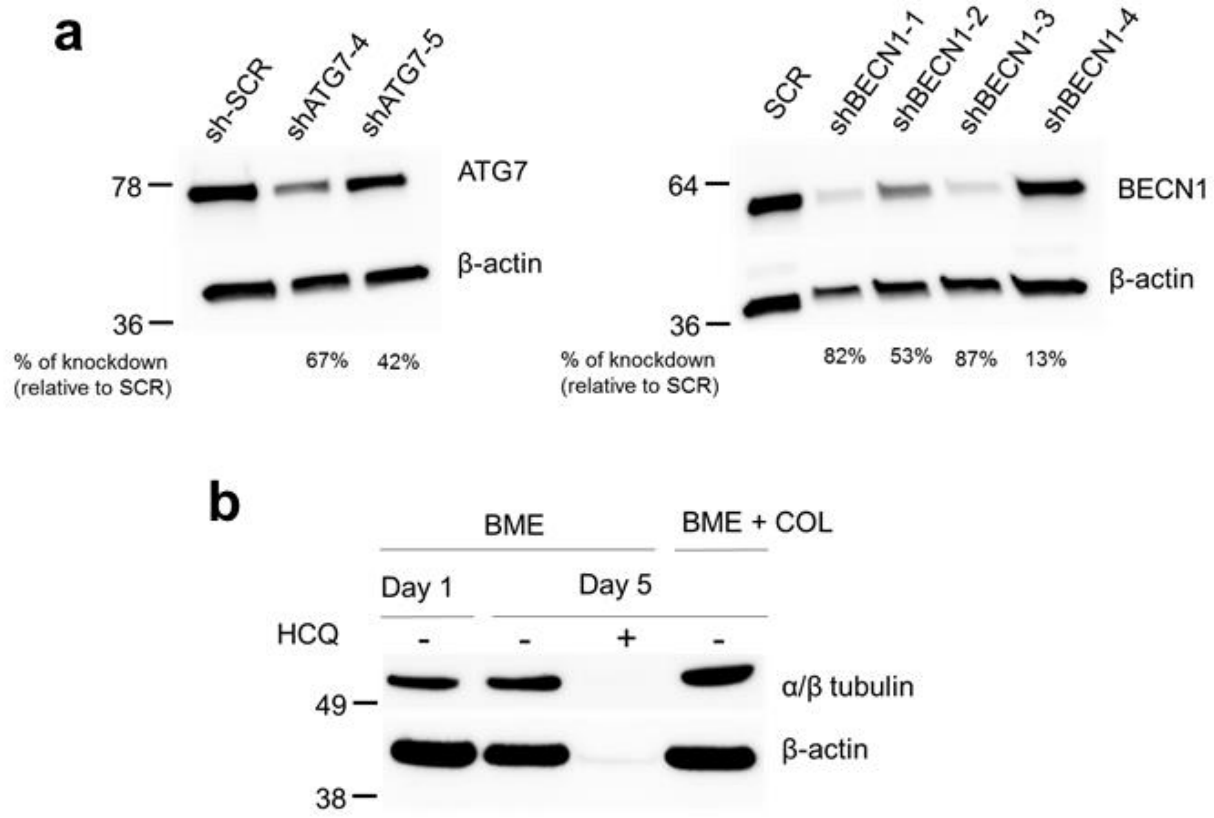

Supplementary Fig. 8. Representative western blots analysis of **a.** reduced ATG7 and BECN1 expression in shRNA clones and **b.** α/β tubulin and β-actin expression in D2.0R cells plated on BME or BME plus COL1 with or without hydroxychloroquine (HCQ) on days 1 and 5. 10 mg of protein loaded in each lane.

Fig. 5c

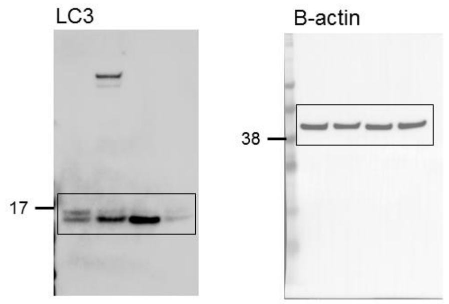

Fig. 6d

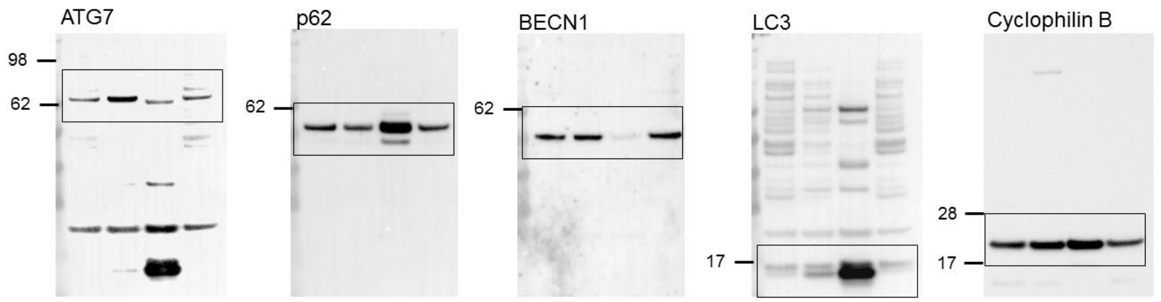

Supplementary Fig. 9. Raw western blot data. Uncropped images of western blots in main figures 5c and 6d are shown.

Fig. 7a

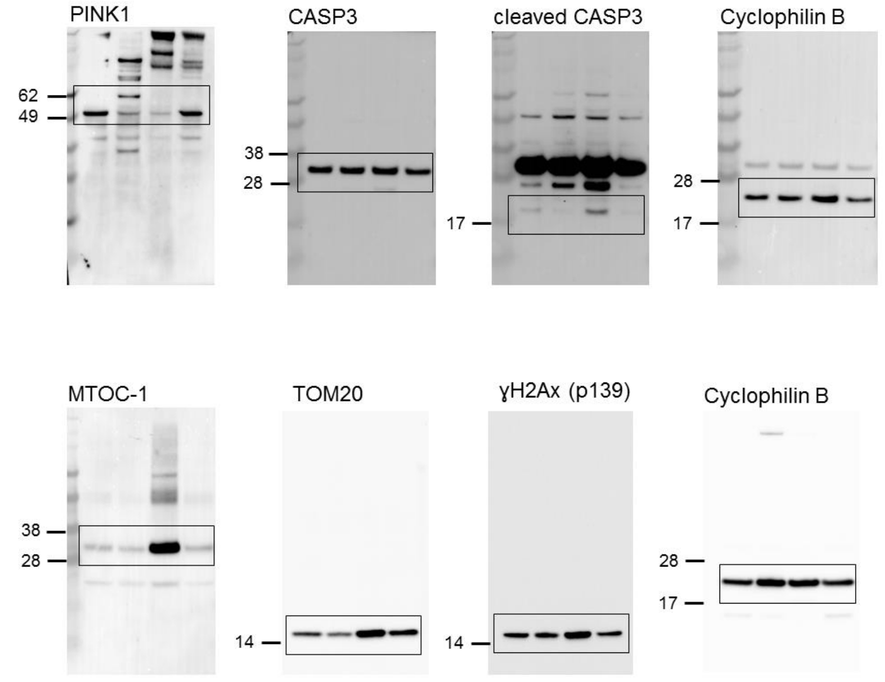

Supplementary Fig. 10. Raw western blot data. Uncropped images of western blots in main figure 7a are shown

Supplementary Table 1 Top 5 canonical pathways upregulated in D2.0R cells on BME vs. BME plus COL1 at day 5 of culture.

Ingenuity Canonical Pathways	*P*-value
Autophagy	2.51189E-11
Colorectal Cancer Metastasis Signaling	6.45654E-10
Mouse Embryonic Stem Cell Pluripotency	1.14815E-07
Pancreatic Adenocarcinoma Signaling	1.31826E-07
Role of Osteoblasts, Osteoclasts and Chondrocytes in Rheumatoid Arthritis	1.7378E-07

Supplementary Table 2 Key autophagy, mitophagy and apoptosis markers differentially modulated in D2.0R cells on BME vs. BME plus COL1 at day 5 of culture.

Gene ID	RPM	FDR step up (BME vs. COL)	Fold change
**Autophagy**			
Sqstm1/p62	3.84E+03	4.25E-06	3.81E+00
Map1lc3b/lc3b	7.37E+02	9.31E-06	2.71E+00
Atg7	1.14E+02	5.45E-05	1.76E+00
Map1lc3a/lc3a	6.42E+01	3.05E-04	2.05E+00
Becn1	4.77E+02	7.53E-02	1.11E+00
**Mitophagy**			
Atp5b	7.88E+03	3.22E-04	-2.06E+00
Pink1	1.38E+02	1.54E-05	4.10E+00
Tomm20	1.78E+03	4.25E-04	-1.70E+00
Vdac1	1.65E+03	1.26E-03	-1.91E+00
Parl	1.66E+02	5.90E-05	-1.82E+00
**Apoptosis**			
Casp3	1.37E+03	5.82E-05	-2.05E+00
Parp1	6.21E+02	2.64E-04	-2.73E+00
Bcl2	9.76E+01	5.30E-04	1.78E+00
Bak1	5.07E+02	3.26E-04	-1.97E+00

RPM, reads per million.
